# Leistungs- und Kostenkalkulation für eine universitäre, augenheilkundliche Hochschulambulanz

**DOI:** 10.1007/s00347-021-01529-8

**Published:** 2021-11-20

**Authors:** C. Framme, M. Dittberner, K. Rohwer-Mensching, J. Gottschling, P. Buley, K. Hufendiek, K. Hufendiek, B. Junker, J. Tode, F. Lammert, I. Volkmann

**Affiliations:** 1 Universitäts-Augenklinik, Medizinische Hochschule Hannover, Carl-Neuberg-Str. 1, 30652 Hannover, Deutschland; 2 TimeElement, Stampfenbachstr. 52/56, 8092 Zürich, Schweiz; 3 Zentrum für Informationsmanagement (ZIMT), Medizinische Hochschule Hannover, Carl-Neuberg-Str. 1, 30652 Hannover, Deutschland; 4 Stabsstelle Klinische Leistungsentwicklung des Vorstands für Krankenversorgung, Medizinische Hochschule Hannover, Carl-Neuberg-Str. 1, 30652 Hannover, Deutschland; 5 Vorstand für Krankenversorgung, Medizinische Hochschule Hannover, Carl-Neuberg-Str. 1, 30652 Hannover, Deutschland

**Keywords:** Fallpauschale, Poliklinik, IS-H/i.s.h.med, TimeElement, Betriebswirtschaft in der Augenheilkunde, Flat rate per case, Outpatient clinic, IS-H/i.s.h.med, TimeElement, Business management in ophthalmology

## Abstract

**Hintergrund:**

Allgemein gelten Hochschulambulanzen in Universitätskliniken als defizitär. In der vorliegenden Publikation wird der Ansatz unternommen, im Sinne einer Kostenträgerrechnung Umsatz und Kosten der Hochschulambulanz der Medizinischen Hochschule Hannover (MHH) zu evaluieren sowie eine Aufstellung der Arbeitsleistung zu unternehmen.

**Material und Methode:**

Mithilfe der Daten des klinikeigenen Informationssystems (SAP) und einer eigenen Software (TimeElement), welche standardisiert angewendet wird, um den Patientenflow in unserer Hochschulambulanz in Echtzeit online zu erfassen, wurden alle Patientenkontakte des Jahres 2019 evaluiert. Die Gesamtkosten summieren sich aus Personal‑, Material- und Raumkosten inklusive Infrastruktur und werden den pauschalierten Erlösen nach Hochschulambulanzvertrag (HSA-Bereich) sowie weiteren Erlösen aus Konsilleistungen, Selbstzahlern, ambulantem Operieren und Kooperationsverträgen für intravitreale Injektionen (IVOMs) gegenübergestellt.

**Ergebnisse:**

Bei durchschnittlich 10,6 assistenzärztlichen und 3,6 fachärztlichen Stellen sowie 21 nichtärztlichen Stellen (plus 4 BUFDIs [Bundesfreiwilligendienst]) in unserer Hochschulambulanz errechnen sich 2.927.022 € Personalkosten inklusive Overhead für das Gesamtjahr. Zuzüglich der Infrastruktur (524.942 €) sowie Material- und Sachkosten einschließlich Overhead sowie interner Leistungsverrechnung (ILV) in Höhe von 258.657 € beliefen sich die Gesamtkosten in 2019 auf 3.710.621 €. Dem standen Einnahmen von 3.524.737 € aus den oben genannten Bereichen gegenüber, womit sich ein Defizit von −185.884 € (5 %) ergab. Auffallend sind die deutlich unzureichende Finanzierung der Hochschulambulanz über die Fallpauschale und die notwendige Querfinanzierung aus den Bereichen des ambulanten Operierens, der IVOMs und der Selbstzahler. Insgesamt kam es zu den regulären Sprechzeiten zu 19.453 Patientenkontakten bei 17.305 abrechenbaren Fällen. Mit *n* = 9943 waren der Großteil der Kontakte HSA-Besuche. Abrechenbare Fälle resultierten daraus allerdings nur in 82 % aufgrund mehrfacher Besuche pro Quartal. Die gesamte Anwesenheit betrug im Median 3,21 h (durchschnittlich 3,38 h). Durchschnittlich wurden 78 Patientenkontakte pro Arbeitstag gezählt. Dabei ergab die Analyse mittels TimeElement im Median pro Patient 2 Arztkontakte (durchschnittlich *n* = 1,91). Die gesamte Dauer ärztlicher Interaktionen betrug im Median 18 min (durchschnittlich 23 min). In der Funktionsdiagnostik zählten wir im Median ebenfalls 2 Interaktionen pro Patient (durchschnittlich *n* = 2,31), wobei die gesamten Interaktionen im Median 18 min dauerten (durchschnittlich 23 min). Insgesamt wurden innerhalb der Funktionsdiagnostik *n* = 37.363 Einzelleistungen im Jahr 2019 gezählt, wobei mit *n* = 10.888 die optische Kohärenztomographie (SD-OCT) die Hauptleistung darstellte.

**Schlussfolgerungen:**

In einer reinen Kosten‑/Umsatz-Rechnung ergibt sich an der MHH ein leicht defizitäres Ergebnis der Hochschulambulanz. Somit erscheinen die Kosten einer universitären, augenheilkundlichen Hochschulambulanz in Niedersachsen durch die direkten ambulanten Einnahmen nicht ausreichend gedeckt zu sein. Eine Beibehaltung von quartalsweisen Fallpauschalen für alle Fälle der Ambulanz würde in unserem Setting eine Honorierung von ca. 214 € notwendig machen, um die Kosten zu decken. Aktuell werden die zu niedrigen Pauschalen im HSA-Bereich von den anderen Bereichen kompensiert. Die hohe medizinische Arbeitsleistung in unserem Setting erfordert einen ebenfalls hohen Personalaufwand mit einem erheblichen Personalkostenanteil von annähernd 80 %.

## Abstract (en)

**Background:**

Outpatient procedures at a university hospital are generally considered to be unprofitable. In the present publication we evaluate the turnover and costs of the university eye outpatient department of the Hannover Medical School (MHH) in terms of a cost unit accounting as well as providing a summary of the workload.

**Material and method:**

Given the data of the hospital information system (IS-H/i.s.h.med from SAP) and a proprietary software (TimeElement), all patient contacts in the year 2019 were evaluated. The latter software is applied in a standardized manner to record the patient flow of our outpatient service in real time electronically. The total costs consist of personnel, material and room costs including infrastructure of the MHH and are compared to the flat-rate revenues according to the university outpatient contract (HSA) as well as further revenues from internal referral services, self-pay patients, outpatient surgery and cooperation contracts for intravitreal injections (IVOM).

**Results:**

With an average full-time equivalent (FTE) headcount of 10.63 assistant physicians, 3.6 specialist physicians, and 21 nonphysicians (plus 4 Federal Volunteer Service, BUFDI) in our policlinic, we have determined € 2,927,022 in personnel costs, including overheads, for the entire year. Including infrastructure (€ 524,942), material and equipment costs with overheads and internal cost allocation of € 258.657, the total costs in 2019 resulted in € 3,710,621. In contrast, the total income in 2019 was € 3,524,737 generated through the abovementioned patient segments, resulting in a deficit of € −185,884 (5%). Our data provide evidence that regular outpatient revenues are insufficient and are mainly balanced by outpatient surgery, IVOMs and self-pay patients.

In total, there were 19,453 patient contacts during regular office hours (with 17,305 billable cases). At *n* = 9943, the majority of the contacts were HSA visits; however, only 82% of the cases could effectively be charged due to multiple visits per quarter. The median total patient attendance was 3.21 h (mean 3.38 h). On average, 78 patient contacts were counted per working day. The analysis with TimeElement unveiled a median of *n* = 2 physician contacts per patient (mean *n* = 1.91). The median duration per interaction with a physician was 17.98 min (mean 23.23 min). For diagnostics, we counted a median of *n* = 2 interactions per patient (mean *n* = 2.31), with an entire interaction lasting a median of 18.30 min (mean 22.60 min). In total *n* = 37,363 individual diagnostic procedures were recorded in 2019, with SD-OCT being the primary procedure at *n* = 10,888.

**Conclusion:**

The cost/turnover calculation showed a marginal financial loss through our ophthalmological outpatient department. Thus, the costs of a university eye outpatient department in Lower Saxony do not seem to be sufficiently covered by direct outpatient revenues. Maintaining quarterly flat rates for all cases of the outpatient department would require a fee of about € 214 in our setting to remain cost neutral. Currently, the lower flat rates in the HSA area are compensated by other areas. Obviously, the high content-related workload in our setting requires a high personnel expenditure with a considerable personnel cost contribution of nearly 80%.

Universitäre, augenheilkundliche Hochschulambulanzen leisten einen erheblichen Beitrag zur medizinischen Grundversorgung. In ihnen werden Patienten nach augenfachärztlicher Überweisung zur weiteren Mitbeurteilung und Therapieplanung bei schwerwiegenden Erkrankungen ebenso behandelt wie Notfallpatienten zur Vermeidung einer Erblindung. Diese Ambulanzen sind das Portal für alle ambulanten und stationären Operationen. Ihre Diagnostik ist teils hoch spezialisiert und anderweitig nicht verfügbar.

Dabei wird die Erlössituation oft als defizitär erachtet. Die Finanzierung wird maßgeblich über standardisierte Vergütungsformen wie EBM (Einheitlicher Bewertungsmaßstab), GÖA (Gebührenordnung für Ärzte), DKG-NT (Deutsche Krankenhausgesellschaft Nebenkostentarif) und HSA-Pauschalen (Hochschulambulanzpauschalen) gedeckt.

Anders als im stationären Bereich mit detaillierten Kostenkalkulationen über die DRGs („diagnosis related groups“, Institut für das Entgeltsystem im Krankenhaus GmbH, Siegburg [InEK]) für jeden einzelnen Fall erfolgen im ambulanten, kassenärztlichen Bereich lediglich die quartalsweisen Abrechnungen über Fallpauschalen, die zum Teil historisch bedingt veranschlagt wurden und nur fraglich einer aktuellen betriebswirtschaftlichen Kalkulation standhalten können. Diesbezüglich mag die Vergütung ambulanter Fälle teilweise im Süden Deutschlands höher als im Norden oder Osten Deutschlands sein. Auch an unserer Klinik betrugen die Pauschalen für Patienten der Hochschulambulanz vor einigen Jahren nur ca. ein Drittel der aktuellen Pauschale von 145,– € pro Patient und Quartal, welche aktuell immer noch nicht auskömmlich ist.

Letztlich verhandelt jedes Uniklinikum für sich, sodass die Pauschalen und die Leistungsbestandteile variieren. Im Gegensatz hierzu ist die Vergütung im stationären Sektor einheitlich geregelt.

Grundsätzlich können die Kosten einer Hochschulambulanz über die Faktoren Raummieten (Strukturkosten inklusive Overhead), Materialkosten und Personalkosten berechnet werden. Aufgrund der dualen Finanzierung der Universitätskliniken werden die Kosten für bauliche Maßnahmen und die primäre Anschaffung von Großgeräten nicht berücksichtigt, da diese durch das Land Niedersachsen getragen werden. Die Ausgaben für Patientenversorgung und Instandhaltung werden über die Kostenträger der Krankenversicherungen bzw. die Patienten selbst finanziert. Somit sind gerade die Personalkosten der wesentliche Kostenfaktor, deren exakte Berechnung einigen Schwierigkeiten unterliegen kann. Beispielsweise kann ein oberärztlicher Dienst nur partiell inkludiert werden, da diese Person anteilig zwar die Hochschulambulanz, aber eben auch Operationssaal und Station einer Klinik variabel bedient.

Interessanterweise ist nun die inhaltliche Arbeitsleistung einer augenheilkundlichen Hochschulambulanz außer im Bereich der Fallanzahl der Patienten nicht wirklich bekannt und unseres Wissens bisher auch nicht detailliert dargestellt worden. Somit gibt es keine Daten darüber, wie viel inhaltliche Arbeitsleistung beispielsweise in der Funktionsdiagnostik steckt, die aus diagnostischer Sicht einen wesentlichen Schlüsselfaktor in der Patientenbehandlung einer Augenambulanz darstellt. Diese kann deutlich ausgeprägter und zeitintensiver sein als in einer Praxis, da in einer Hochschulambulanz die meisten Patienten bereits von fachärztlichen Kolleginnen und Kollegen überwiesen werden und somit die Universitätsklinik oft differenzialdiagnostisch tätig wird. Zusätzlich werden Assistenzärztinnen und -ärzte mittels 4‑Augen-Prinzip im Rahmen der oberärztlichen Patientenvorstellung/Supervision weitergebildet.

Aktuell gibt es noch keine so fein aufgeschlüsselten inhaltlichen und qualitativen Daten darüber, welche und wie viel Arbeitsleistung definitiv benötigt werden. Die MHH verfügt zur Erfüllung ihrer internen und externen Informationspflichten über eine weitreichende und detaillierte Kosten- und Leistungsrechnung. Die Ergebnisse der Kosten- und Leistungsrechnung sind unter anderem Grundlage der Teilnahme der MHH an der InEK-Kalkulation als „kalkulierendes Krankenhaus“ und der Trennungsrechnung gemäß EU-Vorgaben sowie der seit 2016 nach § 63a NHG (Niedersächsisches Hochschulgesetz) durchzuführenden Trennungsrechnung für Forschung und Lehre einerseits und Krankenversorgung andererseits. Dieselbe Kosten- und Leistungsrechnung war die Basis für die Ermittlung der Kosten der Hochschulambulanzen.

Die Besonderheiten in der Arbeitsweise einer augenheilkundlichen Hochschulambulanz hatten wir bereits in unserer Auswertung der reduzierten Arbeitsleistung unserer Hochschulambulanz für die Zeit des COVID-19-Shutdowns im Frühjahr 2020 thematisiert [3]. Über die in unserem Haus verwendete Software „TimeElement“ ist es möglich, sämtliche Patienten der augenheilkundlichen Hochschulambulanz virtuell online zu erfassen und die Patientenwege durch die verschiedenen Untersuchungsstationen innerhalb der Klinik nicht nur detailgetreu im Sinne eines „Patienten-Tracking“ zu verfolgen, sondern auch die internen Überweisungsaufträge in die unterschiedlichen Funktionseinheiten virtuell „per Knopfdruck“ zu generieren und im Zielbereich abzurufen [6]. Wir sind in der Lage, den Patientenweg eines jeden einzelnen Patienten sekundengenau zu erfassen und idealerweise auch Untersuchungs‑/Wege- und Wartezeiten bei Erkennung freier Kapazitäten für einzelne Untersuchungen so gering wie möglich zu halten. Dadurch können wir detailliert die notwendige Arbeitsleistung der einzelnen Berufsgruppen an jedem einzelnen Patienten messen und darstellen.

Ziel dieser Arbeit ist es, die Kosten für das Jahr 2019 (vollständige Arbeitsleistung ohne COVID-Einschränkungen) unserer mittelgroßen universitären Hochschulambulanz aufzuschlüsseln und fallbezogen den Entgelten für die Patientenbehandlung entgegenzustellen. Damit soll die Frage beantwortet werden, ob unsere Hochschulambulanz auskömmlich vergütet wird oder nicht und ob es gerechtfertigt ist, hier finanziell adäquat entgegenzusteuern. Darüber hinaus soll die inhaltliche Arbeitsleistung an Patienten inklusive Arztkontakten und Funktionsdiagnostik analysiert werden, um eine Basis für zukünftige Vergleiche zu generieren, was auch dem Bedürfnis der Bundesärztekammer (BÄK) Rechnung trägt, entsprechende Personalbedarfsplanungen für den ärztlichen Bereich in den Kliniken aufzusetzen [10].

## Material und Methode

In Zusammenarbeit mit administrativen Abteilungen der MHH wurden die für die Leistungsberechnung erforderlichen Daten aus dem internen Controlling-Informationssystem COINS (Controlling-Informations-System, COINS Information Systems AG, Köln), IS-H/i.s.h.med (Cerner Corporation, North Kansas City, MO, USA, und SAP SE, Walldorf) und der in der Augenklinik zusätzlich genutzten Datenbank TimeElement (MHH, Hannover) [6] extrahiert. Der Patientenflow in unserer Klinik wurde bereits zuvor beschrieben [3].

In der Regel werden nahezu alle externen Patienten ambulant über eine Fachärztin oder einen Facharzt für Augenheilkunde unserer Ambulanz zugewiesen. Notfallpatienten werden zu den regulären Sprechzeiten ebenfalls im Rahmen der Hochschulambulanz behandelt. Die reguläre Arbeitszeit besteht von 07.30 bis 16.30 Uhr (Notfälle außerhalb dieser Zeiten und am Wochenende wurden nicht erfasst).

Unsere Hochschulambulanz besteht patientenbasiert aus den 3 Bereichen allgemeine Ambulanz, Poli-OP und „Sehschule“. Der Patientenfluss im Poli-OP und in der Sehschule (inklusive Orthoptisten) variiert von demjenigen der allgemeinen Ambulanz; in der Regel wird aber ebenfalls in beiden Segmenten nach einer assistenzärztlichen Voruntersuchung und einer ggf. nötigen oberärztlichen Supervision der Patient entsprechend behandelt.

Intravitreale Injektionen (IVOMs) erfolgen im Rahmen unseres Treat-and-Extend-Regimes als eigenständiger Workflow [7, 11]. Die Abrechnung der IVOMs erfolgt teilweise über die Hochschulambulanzpauschale und teilweise über Selektivverträge. Ambulante Operationen (Lidchirurgie, Tumorchirurgie, einfache Hornhaut- und Vorderkammereingriffe) erfolgen über unseren Poli-OP. Die Patienten kommen direkt zur Operation und sind bei einem früheren Termin komplett vorbereitet worden. Ambulante Kataraktoperationen finden in einem zentralen Ambulanzoperationszentrum außerhalb unserer Hochschulambulanz statt und fließen nicht in die Kostenkalkulation unserer Hochschulambulanz mit ein. Das Hochschulambulanz-basierte ambulante Operieren (AO) besteht somit aus Lidoperationen, einfachen Hornhaut‑/Vorderkammereingriffen, den ambulanten Lasereingriffen sowie IVOM.

Arbeitsplatzbezogen sind im nichtärztlichen Bereich folgende Stellen besetzt: Anmeldung 4x, Sekretariat 3x, Poli-OP 4x, Funktionsdiagnostik 4,5x, Orthoptisten 3,5x, Sehschule 2x sowie 4 Stellen mit Bundesfreiwilligendienst-Leistenden (BUFDIs). Diese Stellen werden mit den entsprechenden Personalkosten je Dienstart (Arbeitgeber brutto; „Normkosten“) [9] multipliziert, sodass daraus die Gesamtkosten für das Jahr 2019 resultieren. In diesem Stellenbudget sind Urlaube und sonstige Fehlzeiten bereits inkludiert. Im Assistenzarztbereich kalkulieren wir mit durchschnittlich 8,5 Ärztinnen und Ärzten, die zur Kernarbeitszeit in der Hochschulambulanz anwesend sind. Hier findet der Ausfall durch Urlaub und Dienste durch die Anrechnung eines Faktors von 1,25 statt, sodass mit 10,63 assistenzärztlichen Stellen gerechnet wird, für die nach durchschnittlich Ä1 (3. Jahr) pro Person 81.636,– € Normkosten [9] entstehen. Von diesen sind durchschnittlich 4 Assistenzärzte in der allgemeinen Sprechstunde und 1 bis 2 Assistenzärzte in der Sehschule, 1 Assistentsarzt im Privatbereich sowie 1 bis 2 Assistenzärzte im Poli-OP-Bereich inklusive entsprechender Voruntersuchungen eingesetzt. Zusätzlich rechnen wir über den gesamten Hochschulambulanzkomplex mit 2,6 Oberärzten (Ä32) und 1 Facharzt (Ä21), bei denen gleichermaßen der Faktor 1,25 zum Ausgleich von Fehlzeiten anzusetzen ist.

Die Kosten für Flächenbewirtschaftung sowie Materialkosten können dezidiert über das klinikeigene COINS-System extrahiert werden. Für die Flächenbewirtschaftung wird eine Kaltmiete zuzüglich der Nebenkosten erhoben, die sich an der DIN-Norm DIN 277-1:2016-01 orientieren. Die von den Ambulanzen genutzten Räume sind mit ihren Flächen der Kostenstelle der jeweiligen Ambulanz zugeordnet. Damit werden auch die Bewirtschaftungskosten dieser Fläche exakt den ambulanten Kostenstellen zugeordnet. In die Nebenkosten fließen die Positionen Reinigung, Energie, Technik, Ver- und Entsorgung sowie Objektbetreuung anteilig der jeweiligen Flächenarten und Mietklassen ein.

Während über das SAP-System alle Kontakte abrufbar sind, wurden über TimeElement alle Kontakte im HSA-Bereich, im Selbstzahlerbereich, im Sehschulbereich, jedoch aufgrund der hohen Taktung nicht im IVOM-Bereich mit unserem individualisierten Treat-and-Extend-System [5] sowie im AO gezählt.

## Ergebnisse

Die oben aufgeführten Segmente liefern nach Tab. 1 folgende Gesamtkosten im Bereich Personal (Tab. 1): Im nichtärztlichen Bereich betragen die direkten Kosten insgesamt 1.155.318,– €, im ärztlichen Bereich müssen 1.412.245,50 € kalkuliert werden. Mit dem entsprechenden Overhead summieren sich die Gesamtpersonalkosten für die Hochschulambulanz der MHH im Jahr 2019 im Sinne einer Kostenträgerrechnung auf 2.927.022,39 €. Bezüglich der Raummieten inklusive Bewirtschaftung sind für eine vorhandene Fläche von 1080,20 m^2^ Gesamtkosten von 524.942,– € hinterlegt. Die Material- und Sachkosten der Hochschulambulanz betrugen in 2019 für Wirtschafts- und Verwaltungsbedarf, zentrale Dienstleistungen, Instandhaltung, medizinischen Bedarf (Medikamente, IVOM-Sets, Verbandsmaterial, Desinfektion etc.) 148.568,– €. Zusätzlich fielen Kosten von 78.324,– € für die interne Leistungsverrechnung (ILV) der von der Hochschulambulanz bezogenen Leistungen an. Auch für die Material- und Sachkosten sowie die ILV muss der Overhead verrechnet werden, sodass für diese Posten insgesamt 258.656,88 € veranschlagt werden. Die Gesamtkosten unserer Hochschulambulanz beliefen sich damit im Jahr 2019 auf 3.710.621,27 €.

**Table 1:** 

Tarifgruppe	VK	Normkosten	∑ PK
E6	13	50.568,00 €	657.384,00 €
E8	4,5	54.816,00 €	246.672,00 €
E9	3,5	63.012,00 €	220.542,00 €
BUFDI	4	7680,00 €	30.720,00 €
*Gesamt*	*25*		*1.155.318,00* *€*
AE13	8,5	81.636,00 €	693.906,00 €
AE21	1	98.088,00 €	98.088,00 €
AE32	2,6	129.924,00 €	337.802,40 €
*Gesamt*	*12,1*		*1.129.796,40* *€*
**Ausfallfaktor**		**1,25**	**1.412.245,50** **€**
Gesamtkosten			2.567.563,50 €
OH		14,00 %	359.458,89 €
*Personalkosten Summe*			*2.927.022,39* *€*

Der ärztliche Ausfallfaktor von 1,25 bildet Ausfälle durch Urlaub, Fortbildung, Krankheit und Dienstfrei nach Nachtdiensten ab

Das Informationssystem COINS weist für 2019 unter dem Punkt „Erlöse aus Krankenhausambulanz“ 2.679.848,– € ohne ambulante Kataraktoperationen aus. Darin enthalten sind neben den HSA-Einnahmen maßgeblich Erlöse aus „ambulantem Operieren“ (210.469,– €) und den IVOMs aus Selektivverträgen (inklusive OCTs = 546.002,– €). Inklusive weiterer Erlöse für privatärztliche IVOMs und weitere ambulante Privatbehandlungen (679.730,– €) sowie Konsiltätigkeiten (165.159,– €) summiert sich der Erlösanteil insgesamt auf 3.524.737,– €. Damit ergibt sich rechnerisch ein Defizit in der Bilanz für unsere Hochschulambulanz von −185.884,– €. Dieses entspricht 5 % der Gesamtkosten.

Insgesamt kam es zu den regulären Sprechzeiten der Poliklinik in 2019 zu 19.453 Kontakten bei 17.305 abrechenbaren Fällen. Im Notfallbetrieb außerhalb der regulären Sprechzeiten und am Wochenende wurden zusätzliche 1914 Kontakte gezählt, die hier nicht weiterverfolgt werden.

Heruntergebrochen auf die Fallzahl im Regelbetrieb der Hochschulambulanz (HSA-Bereich) müsste eine Quartalspauschale von 214,42 € pro Patient erlöst werden (statt aktuell 145,– €), um die Gesamtkosten ohne Querfinanzierung auszugleichen. Mit *n* = 9943 waren ein Großteil der Kontakte HSA-Besuche. Abrechenbare Fälle resultierten daraus allerdings nur in *n* = 8170 (82,17 %) mit einem Erlös von 1.184.650,– €. Die Tab. 2 zeigt, dass lediglich 85,2 % der Fälle nur 1 Besuch pro Quartal aufweisen, nahezu 15 % aber 2 oder mehr Besuche, die nicht vergütet werden, aber in der Regel ähnliche Leistungen benötigen (Tab. 2). Würde man diese Besuche ebenfalls mit der aktuell gültigen Pauschale von 145,– € vergüten, so wäre hier ein weiterer Erlös durch Addition der weiteren Besuche (Tab. 2) von 267.815,– € vorhanden. Somit wird die Leistung im HSA-Bereich zu 18,37 % nicht vergütet.

**Table 2:** 

Anzahl Besuche	Anzahl Fälle	In % alle Fälle
1	6962	85,21
2	796	9,74
3	286	3,50
4	84	1,03
5	19	0,23
6	14	0,17
7	3	0,04
8	1	0,01
9	1	0,01
10	1	0,01
12	2	0,02
18	1	0,01
*Gesamtergebnis*	*8170*	*100,00*

Zählt man die anderen Patientenkontakte (s. oben) abseits des HSA-Bereichs (*n* = 9510) und berechnet den Erlöswert pro Kontakt, so ergibt sich aus diesen Einnahmen von 2.340.087,– € ein „Kontaktwert“ von 246,06 €. Übertragen auf Fälle (*n* = 7362) entspricht dieses einem Fallwert von 317,86 €. Aus dieser Diskrepanz wird ersichtlich, dass der HSA-Bereich maßgeblich von den anderen Bereichen wie dem Privatsegment, den IVOMs und dem ambulanten Operieren mitfinanziert wird (und dennoch nicht auskömmlich beschieden ist!).

Durch eine kontaktbezogene Pauschale und nicht durch eine quartalsbezogene Pauschale (+267.815,– € [s. oben]) würde sich das Gesamtdefizit von −185.884,– € auf ein Plus von +81.931,– € bewegen. Folglich wäre bei kontaktbezogener Honorierung im HSA-Bereich (*n* = 9943 Kontakte [s. oben]) eine Fallpauschale von 137,84 € für eine auskömmliche Finanzierung notwendig unter der Voraussetzung, dass die HSA-Kompensation durch die anderen Bereiche im Sinne einer „schwarzen Null“ gleichbleiben würde. Bei den Kostenanalysen ist allerdings auch zu beachten, dass im Folgejahr 2020 personelle Mehrkosten (Erhöhung der Normkosten z. B. durch Lohnerhöhungen bei gleicher Anzahl der Mitarbeiter) von 93.684,06 € auftraten, sodass dieses Plus wieder aufgehoben wäre.

Die Analyse der Arbeitsleistung ergab durchschnittlich 77,5 Patienten pro Tag (SAP-Datenbank) bei 251 Arbeitstagen im Jahr 2019. Dabei wurden durchschnittlich pro Tag 13,64 IVOMs (*n* = 3426 IVOMs; privat: *n* = 391; Selektivvertrag: *n* = 1692, Kassenbereich: *n* = 1343) durchgeführt. Im Bereich AO wurden *n* = 1862 Operationen gezählt, von denen 598 Eingriffe im Poli-OP erfolgten (Durchschnittswert pro Arbeitstag: *n* = 7,42). Somit wurden täglich 56,44 Kontakte über Sehschule und Allgemeinambulanz behandelt.

Bei Gesamtjahreskosten von 3.710.621,27 € kostete jeder Ambulanztag 14.783,35 €. Dieses bedeutet 190,75 € Kosten pro Patient. Bezüglich der reinen Personalkosten von 2.927.022,39 € kostete jeder Ambulanztag 11.661,44 € (150,47 € pro Patient). Insgesamt waren 78,9 % der Gesamtkosten reine Personalkosten. Dieses zeigt, wie personalintensiv eine augenheilkundliche Hochschulambulanz in einem universitären Setting aufgestellt ist und dass die Fallpauschalen im HSA-Bereich deutlich zu niedrig kalkuliert sind.

**Figure 1:**
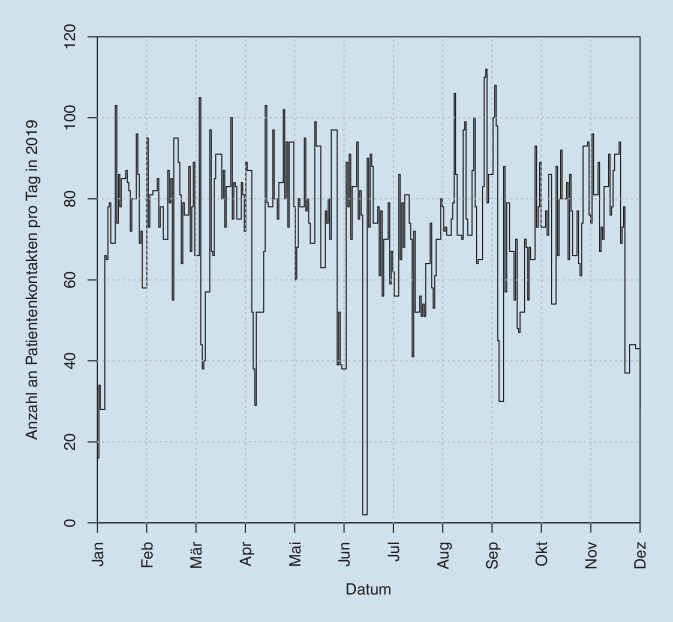

**Figure 2:**
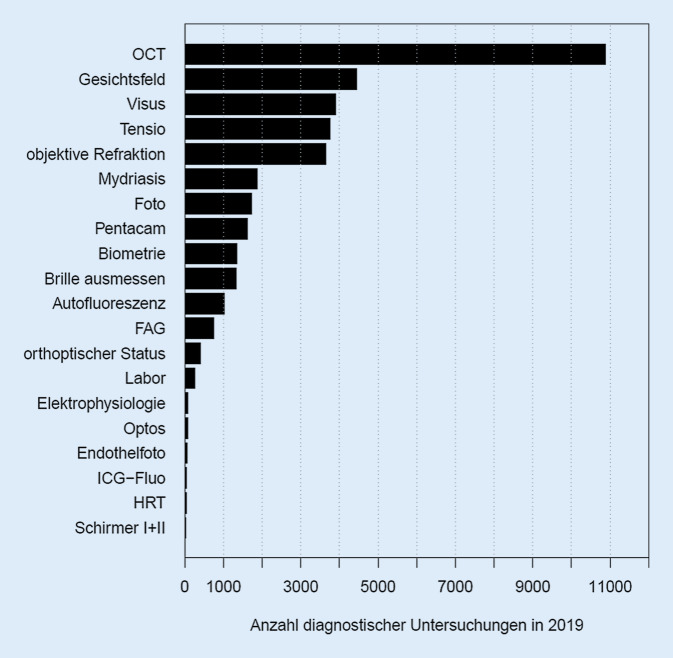

Die Abb. 1 zeigt die entsprechende Fluktuation der Kontaktdaten über das Jahr 2019 (Abb. 1). Die Evaluation unserer Daten über TimeElement ergab eine Kontaktanzahl von durchschnittlich *n* = 73,6 pro Tag, die damit etwas niedriger lag als bei SAP. Dabei ergaben sich in den Messungen mittels TimeElement im Median pro Patient 2 Arztkontakte (durchschnittlich *n* = 1,91). Die Dauer der ärztlichen Interaktionen pro Patient betrug im Median 17,98 min (durchschnittlich 23,23 min) (Tab. 3). Bezüglich Diagnostik zählten wir im Median ebenfalls 2 Interaktionen pro Patient in der Funktionsdiagnostik (durchschnittlich *n* = 2,31), wobei die Gesamtinteraktionen im Median 18,30 min dauerte (durchschnittlich 22,60 min; Tab. 3). Die Tab. 4 weist die ärztlichen Konsultationszeiten vom ersten Patientenkontakt (Assistenz) und zweitem Kontakt (Supervision) aus, wobei der Erstkontakt in der Regel geringfügig länger dauerte (Tab. 4). Die Abb. 2 zeigt die Verteilung der einzelnen diagnostischen Maßnahmen, wobei die SD-OCT den größten Stellenwert besitzt (Abb. 2). Insgesamt wurden in unserer Funktionsdiagnostik *n* = 37.363 Einzelleistungen im Jahr 2019 gezählt. Könnte man beispielsweise für jede der insgesamt *n* = 10.888 in der Funktionsdiagnostik durchgeführten SD-OCT-Untersuchungen (und hier sind die IVOM-spezifischen SD-OCTs nicht enthalten!) die veranschlagten 93,84 € als Einzelleistung nach GOÄ (2,3-fach) [1] liquidieren, so würden dadurch zusätzlich 1.021.730,– € erlöst werden, was bereits mehr als 25 % der Gesamtkosten kompensierte.

**Table 3:** 

	Ärztlich	Diagnostisch	Total
*Min*	3,02	3,02	3,02
*25-%-Quantil*	10,29	7,97	13,90
*Median*	17,98	18,30	26,75
*Mittel*	23,23	22,60	32,55
*75-%-Quantil*	30,17	31,43	43,88
*Max*	119,05	105,75	190,77
*SD*	18,37	18,19	25,01

*SD* Standardabweichung vom Mittelwert

**Table 4:** 

	Behandlungszeit erster Patientenkontakt in min	Behandlungszeit zweiter Patientenkontakt in min
*Min*	3,02	3,02
*25-%-Quantil*	7,82	6,55
*Median*	11,60	9,90
*Mittel*	13,70	12,79
*75-%-Quantil*	17,32	15,38
*Max*	73,88	74,05
*SD*	8,51	9,76

*SD* Standardabweichung vom Mittelwert

**Figure 3:**
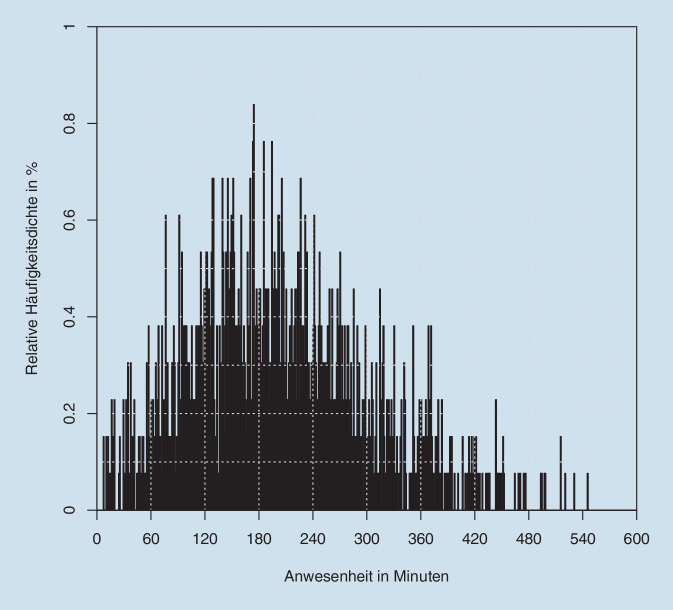

Die gesamte Anwesenheitszeit von Patienten in unserer Hochschulambulanz war Fall- und Wartezeit-bezogen sehr heterogen und betrug im Median 3,21 h (im Durchschnitt 3,38 h, Tab. 5). Diese heterogene Verteilung ist in Abb. 3 dargestellt.

**Table 5:** 

	Minuten	Stunden
*Min*	7,38	0,12
*25-%-Quantil*	128,23	2,14
*Median*	192,63	3,21
*Mittel*	202,56	3,38
*75-%-Quantil*	266,53	4,44
*Max*	545,95	9,10
*SD*	99,63	1,66

*SD* Standardabweichung vom Mittelwert

## Diskussion

In dieser Analyse wird erstmals der Versuch unternommen, im Rahmen einer entsprechenden Berechnung zu evaluieren, inwieweit sich eine Hochschulambulanz in der Augenheilkunde finanziell selbstständig trägt. Die anfangs getroffene Annahme, dass eine universitäre, augenheilkundliche Hochschulambulanz in Deutschland finanziell dabei eher unterfinanziert ist, hat sich in unserer Kosten‑/Erlös-Berechnung bestätigt. Das Defizit für die gesamte Hochschulambulanz beträgt bei uns innerhalb der dargestellten Rahmenbedingungen ca. 5 %, was sich prinzipiell noch nach einem verhältnismäßig niedrigen „Minus“ anhört. Dieses geringere Defizit gelingt aber nur durch die Querfinanzierung der reinen Fallpauschale durch die Bereiche AO, IVOM und Selbstzahler. Hier wird deutlich, wie unzureichend die Vergütung der Fallpauschale ist (Fallpauschale HSA 145,– € vs. Fallwert AO/Selbstzahler 317,86 €).

Im Gegensatz zu den stationären Kostenträgerberechnungen des DRG-Systems gibt es im ambulanten Bereich mit seinen Fallpauschalen keine belastbaren Kostenkalkulationen [8]. Wie bereits für strabologische Operationen im ambulanten Setting eine deutliche Unterfinanzierung auf universitärer Ebene dargestellt werden konnte [4], zeigt auch diese Untersuchung auf, dass der HSA-Bereich mit seiner aktuellen Pauschale an der MHH von 145,– € pro Fall pro Quartal deutlich unterfinanziert ist und für eine adäquate Deckung der Kosten des HSA-Bereichs in unserem Setting 214,– € Quartalspauschale pro Fall veranschlagt werden müssten. Lediglich durch die Gegenfinanzierung dieser zu geringen HSA-Vergütung durch die Bereiche der Selbstzahler, der IVOMs und des ambulanten Operierens (AO) entsteht letztendlich ein relativ kleines Gesamtdefizit.

Der poliklinische Bereich einer Augenklinik ist sehr personalintensiv und umfasst verschiedene Verwaltungsbereiche (Ambulanz, Sehschule, Privatbereich), Orthoptistinnen für die Sehschule, MFA und Pflege für den Bereich AO und Fotografen für die Funktionsdiagnostik. Hinzu kommt der Bereich der Assistenzarztweiterbildung, der ein Vier-Augen-System mit fachärztlicher Supervision erfordert. Insbesondere für die oftmals erforderliche, spezialisierte Diagnostik und Versorgung ist diese umfangreiche Besetzung erforderlich. Bezüglich der Personalkosten erscheint der Kostensatz von knapp 80 % der Gesamtkosten relativ hoch, was sich aber durch die oben dargestellten Besonderheiten und den Umstand, dass Investitionen keine Berücksichtigung finden, erklärt. Da die Hochschulambulanz einer Universität andererseits in ein kostenintensives Umfeld eingebettet ist (Hochschulverwaltung, Gebäudemanagement, Infrastruktur etc.), müssen diese Overheads den Kostenberechnungen aufgeschlagen werden. Dieses ist in der Evaluation in adäquater Weise geschehen. Was – wie oben bereits auch dargestellt – nicht in die Berechnung einfließt, sind die Gerätekosten bei entsprechenden Neuanschaffungen.

Ein unseres Erachtens interessanter Aspekt ist die Tatsache, dass ca. 18 % aller HSA-Patientenkontakte nicht bezahlt werden, da diese Patienten mindestens 2‑mal oder häufiger pro Quartal erscheinen. Würde – wie oben dargestellt – jeder Patientenkontakt unabhängig vom Quartal vergütet werden, so hätte sich das bei uns errechnete Defizit von ca. 185.000,– € in ein Plus von ca. 82.000,– € gewandelt. Eine Vergütung durch Kontaktpauschalen wird durchaus kontrovers diskutiert, da dadurch unerwünschte Mengenausweitungen und Kostensteigerungen zu erwarten wären. Auf der anderen Seite kann allerdings konstatiert werden, dass auch die Hochschulambulanzen in der Regel längere Wartezeiten für Termine haben, sodass diesbezüglich eine weitere Mengenausbreitung bei eher starren Ressourcen schwierig erscheint.

Ein weiterer Aspekt, der in unserem Setting bisher nicht adäquat berücksichtigt wird, ist der Umstand, dass die Kinderaugenheilkunde mit der Strabologie im Gegensatz zu anderen „Spezial-Kinderfachabteilungen“ bei uns nicht vom Kinderzuschlag profitiert. Dies würde Mehreinnahmen von ca. 170.000,– € bei etwa 2000 Kinderkontakten (Alter: 0 bis 18 Jahre) bewirken. Nach SGB V heißt es: „(1a) Ergänzend zur Vergütung nach Absatz 1 sollen die Landesverbände der Krankenkassen und die Ersatzkassen gemeinsam und einheitlich für die in kinder- und jugendmedizinischen, kinderchirurgischen und kinderorthopädischen sowie insbesondere pädaudiologischen und kinderradiologischen Fachabteilungen von Krankenhäusern erbrachten ambulanten Leistungen mit dem Krankenhausträger fall- oder einrichtungsbezogene Pauschalen vereinbaren, wenn diese erforderlich sind, um die Behandlung von Kindern und Jugendlichen, die auf Überweisung erfolgt, angemessen zu vergüten.“ [2] Da die „Kinderaugenheilkunde“ im engeren Sinne in der Regel nicht als eigene „Fachabteilung“ eines Klinikums fungiert und sich die Kassen in Verhandlungen offensichtlich häufig auf diese explizite Regelung berufen, war es in unserer Klinik bisher nicht möglich, den entsprechenden Kinderzuschlag von 100,– € pro Fall (im Rahmen der HSA-Pauschalisierung) zu vereinbaren. Angesichts der Tatsache, dass die Kinderaugenheilkunde insbesondere in den Sehschulen von Universitätskliniken und großen Krankenhäusern eine hoch spezialisierte Sonderstellung einnimmt und in kleineren Settings entsprechende Diagnostik und Therapie in geeigneter Form teilweise gar nicht angeboten werden können, darf es erstaunen, dass hier der Schwerezuschlag nicht ermöglicht wird. Dieses adressiert die gleiche Problematik, die wir bereits bei der operativen Strabologie mit den entsprechenden Mindervergütungen im ambulanten Setting darstellen konnten [4], und birgt die Gefahr, dass spezialisiertes Wissen aufgrund unzureichender Finanzierung und somit fehlenden Anreizes immer weiter verschwindet.

Die inhaltliche Bewertung unserer Arbeitsleistung ist über TimeElement möglich. Hier war es uns wichtig darzustellen, wie viele Patienten täglich „getrackt“ werden, wie lange die Anwesenheitszeiten in unserer Klinik sind und wie viele Untersuchungen inklusive Funktionsdiagnostik und ärztlicher Kontakte wie lange durchgeführt wurden. Die wesentlichen Ergebnisse wie eine relativ lange Anwesenheitszeit von ca. 3,5 h, im Median 2 Arztkontakte pro Patient und ebenfalls 2 funktionsdiagnostische Untersuchungen pro Patient waren zu erwarten, wobei im Rahmen des COVID-Lockdowns im Frühjahr 2020 die Anwesenheitszeiten aufgrund der deutlich reduzierten Patientenkontakte signifikant gesenkt wurden [3]. Dieses lässt bei den in der Regel vielen parallel laufenden Sprechstunden in einer Hochschulambulanz den „Wettbewerb“ der Patienten um funktionsdiagnostische Kapazitäten, aber auch um die oberärztliche Supervision erkennen. Diese Kapazitäten sind durch strukturelle Ressourcen (z. B. SD-OCT-Geräte) und v. a. auch durch personelle Ressourcen (Fotografen zur Gerätebedienung, Patienten pro Assistent sowie Oberärzte zur Supervision) begrenzt.

Krankheitsbedingte Ausfälle sind ein wesentlicher Punkt, arbeitsplatzbezogene Mitarbeitermodelle mit ausreichend „Kompensation“ zu erzeugen, wie es aktuell auch das Ansinnen der Bundesärztekammer ist. Hierfür hat die Deutsche Ophthalmologische Gesellschaft (DOG) einen entsprechenden Arbeitskreis eingerichtet [10]. Unsere Berechnung zielt dabei auf einen ärztlichen Ausfallfaktor von 1,25 (in der Publikation von Schargus et al. wird sogar ein Faktor von 1,3 diskutiert [10]), der entsprechend Urlaub, Fortbildung, Krankheit und insbesondere auch Abwesenheit nach Nachtdienst adressiert.

Am Beispiel unserer 4 SD-OCT-Geräte mit *n* = 37.363 Untersuchungen zeigt sich, dass ausreichend strukturelle Ressourcen erforderlich sind, um Engpässe zu vermeiden. Bei 4 Geräten mit 37.363 Untersuchungen ergeben sich 37,21 Untersuchungen pro Tag und Gerät. Gerade in Spitzenzeiten sind diese Geräte im Dauereinsatz. Am Beispiel unserer IVOM-Sprechstunde wurde deutlich, dass ein zusätzliches Gerät den Gesamtworkflow bei gleicher Personalstärke deutlich beschleunigen konnte [5].

Die vorgelegte Analyse erhebt keinen Anspruch auf Vollständigkeit. Es ist der Versuch, eine möglicherweise auch sehr heterogene Datenlage in einem ambulanten Setting über 2 verschiedene Datenbanken zu evaluieren. Dabei wollen wir sowohl die Kostenrechnung (SAP/COINS) als auch die Arbeitsleistung (TimeElement) verdeutlichen. Nicht berücksichtigt werden z. B. anfallende Überstunden durch das Schreiben von Arztbriefen. Solche Mehrarbeit erhöht das Defizit der Hochschulambulanz noch weiter. Zudem ist die Hochschulambulanzpauschale länderabhängig und kann in anderen Kliniken höher sein. Deshalb behalten unsere Daten trotzdem ihre Relevanz, wir legen mit unserer ausführlichen Analyse eine Basis, wie hoch eine auskömmliche Fallpauschale mindestens sein müsste.

Wir konnten insgesamt anhand einer eigenen unseres Erachtens recht umfassenden Datenanalyse zeigen, dass eine universitäre Hochschulambulanz, wie initial vermutet, in der Tat defizitär ist, wobei es uns positiv überrascht hat, dass das Minus mit 5 % noch relativ niedrig ausgefallen ist. Dennoch fällt auf, dass die HSA-Pauschalen deutlich zu niedrig angesetzt sind und nur partiell durch Nicht-HSA-Komponenten wie ambulantes Operieren oder IVOMs kompensiert werden können. Möglicherweise sind hier sogar auch eher refraktiv starke Polikliniken im Vorteil, die einen höheren „Out-of-pocket“-Anteil für beispielsweise (Laser‑)Chirurgie generieren. Andererseits wäre es bei vorhandenen Ressourcen auch möglich, den Anteil an Untersuchungskabinen (mit entsprechendem ärztlichen Personal) zu erhöhen, wobei damit zumindest die Fixkosten für die bewirtschaftete Fläche stabil blieben, aber Personalkosten steigen und der Wettbewerb von mehr Patienten um die Funktionsdiagnostik und oberärztliche Abnahme zu längeren Anwesenheitszeiten führen würden. Dadurch oder aber auch durch eine Erhöhung der Patientenzahlen pro Assistenzarzt könnte ebenfalls der Umsatz gesteigert werden. Allerdings ist die zeitliche Arbeit der Weiterbildungsassistentinnen und -assistenten im Rahmen der Gesamtorganisation am Patienten zum Teil so hoch (Anamnese, Untersuchung, OA-Vorstellung, funktionsdiagnostische Vorbereitung, Aufklärung zur Angiographie, Aufklärung zur Operation, Verweilkanülen, Blutentnahmen, Rezepte, Kurzbriefe etc.), dass eine gewisse Anzahl von unseres Erachtens ca. 12 bis 16 Patienten pro Tag und je nach Art der Erkrankung zumindest nicht in unserem Setting überschritten werden sollte.

## Fazit für die Praxis

- Als Quintessenz bleibt festzuhalten, dass eine Hochschulambulanz durch die reine Fallpauschale aktuell drastisch unterfinanziert ist. Aus Sicht der Kliniken ist eine adäquate Kontaktpauschale wünschenswert, die nach der vorliegenden Evaluation ca. 191,– € pro Kontakt betragen müsste, um die Hochschulambulanz gewinnneutral zu betreiben. Ein Festhalten an der Quartalspauschale würde unter der Voraussetzung, dass alle Fälle der Ambulanz über Pauschale vergütet würden, derzeit 214,– € bedingen. Daraus, dass der HSA-Anteil in unserer Berechnung 47 % aller Fälle ausmacht, aus diesem Bereich aber nur 33 % aller Einnahmen generiert werden, ist auch nochmals gut ersichtlich, dass hier eben eine deutliche Unterfinanzierung vorliegt.
- Wichtig ist zudem die angemessene Honorierung der Kinderaugenheilkunde durch das entsprechende Zusatzentgelt. Die vorgeschlagenen Maßnahmen würden eine moderate Verbesserung der Finanzierungssituation der Ambulanz erwirken und könnten mehr Möglichkeiten einer verbesserten „Mitarbeiterdecke“ generieren, die in unserer Hochschulambulanz eigentlich trotz personalintensiver Kosten dringend nötig wäre.
- Die Hochschulambulanz ist das Tor in die klinische Versorgung. Wir behandeln dort Problemfälle im ambulanten Setting und rekrutieren komplexe Fälle für eine stationäre Behandlung. Unseres Erachtens müssen die ambulante Leistung und Expertise adäquat honoriert werden, insbesondere da der ambulante Anteil nicht nur generell, sondern auch in vielen anderen Fächern zukünftig eine stärkere Gewichtung erfahren wird und sich somit auch unabhängig von einer stationären Versorgungseinheit selbstständig tragen muss.
